# Hand Book of Practical Obstetrics, Including Anæsthesia

**Published:** 1871-10

**Authors:** 


*Hand Book of Practical Obstetrics, including Anæsthesia.* By John Tanner, M.D. J. B. Lippincott & Co., Philadelphia. J. & A. Churchill, London, 1871.

The Author of this book claims to afford the Accoucheur a complete guide and hand-book of Midwifery, and says that it contains everything of importance that is to be found in other books upon the subject.

The little work seems to us to be well adapted to the wants of the student while attending lectures upon this subject; and also capable of refreshing the minds of all who may require such aid. The illustrations are excellent and numerous, and constitute an attraction in the work. We think from our hasty examination that it has merits which will be fully appreciated, especially by students and young practitioners.

